# Overview of the Association Between the Pathophysiology, Types, and Management of Sickle Cell Disease and Stroke

**DOI:** 10.7759/cureus.50577

**Published:** 2023-12-15

**Authors:** Faisal Hakami, Essam Alhazmi, Wafa M Busayli, Sultan Althurwi, Abdulrahman M Darraj, Mohammed A Alamir, Alyaj Hakami, Renad A Othman, Amal I Moafa, Hassan A Mahasi, Mohammed Ali Madkhali

**Affiliations:** 1 Medicine, Faculty of Medicine, Jazan University, Jazan, SAU; 2 Medicine, Faculty of Medicine, Jazan University, Jizan, SAU; 3 Internal Medicine, and Hematology and Oncology, Faculty of Medicine, Jazan University, Jazan, SAU

**Keywords:** hemoglobin s, hemoglobinopathy, hemorrhagic stroke, ischemic stroke, sickle cell disease

## Abstract

Sickle cell disease (SCD) is a genetic blood disorder that affects hemoglobin and increases stroke risk, particularly in childhood. This review examines the pathophysiological association between SCD and stroke, the classification of stroke types, risk factors, diagnosis, management, prevention, and prognosis. A comprehensive literature search was conducted via PubMed, Scopus, and Cochrane databases. Relevant studies on SCD and stroke pathophysiology, classification, epidemiology, diagnosis, treatment, and prevention were identified.

Sickle cell disease causes red blood cells to become rigid and sickle-shaped, obstructing blood vessels. Recurrent sickling alters cerebral blood flow and damages vessel walls, often leading to ischemic or hemorrhagic strokes (HS). These occur most frequently in childhood, with ischemic strokes (IS) being more common. Key risk factors include a prior transient ischemic attack (TIA), low hemoglobin, and a high leukocyte count. Neuroimaging is essential for diagnosis and determining stroke type. Primary prevention centers on blood transfusions and hydroxyurea for those at high risk. Acute treatment involves promptly restoring blood flow and managing complications. However, significant knowledge gaps remain regarding stroke mechanisms, optimizing screening protocols, and improving long-term outcomes. This review synthesizes current evidence on SCD and stroke to highlight opportunities for further research and standardizing care protocols across institutions. Ultimately, a holistic perspective is critical for mitigating the high risk of debilitating strokes in this vulnerable patient population.

## Introduction and background

Sickle cell disease (SCD) poses a major clinical challenge due to its hematological and neurological complications, like stroke [1]. The link between SCD and stroke is established; approximately 11% of SCD patients have a stroke by age 20 [2]. This high risk stems from SCD's pathophysiology, including hemolysis, vaso-occlusion, and inflammation [3]. Together, these alter cerebral blood flow and promote vasculopathy, often causing ischemic strokes (IS) or hemorrhagic strokes (HS) [4].

Ischemic strokes typically result from cerebral artery narrowing, while HS is associated with vessel rupture [5,6]. For prevention, hydroxyurea and transfusions are commonly used [7]. For treatment, guidelines recommend prompt transfusions, anticoagulants, and sometimes surgery [8].

Since Platt et al.'s landmark study, research has detailed the mechanisms relating SCD to stroke. Hemolysis contributes to vascular dysfunction [2,9], while inflammation promotes cerebrovascular complications [10]. Classifying strokes in SCD as ischemic or hemorrhagic is essential. Adams et al. demonstrated transcranial Doppler's utility in predicting pediatric IS [11]. DeBaun et al. reviewed managing both stroke types, emphasizing transfusion and anticoagulation [6,12].

Despite preventative measures like hydroxyurea and transfusion, knowledge gaps remain [7]. Emerging therapies like gene editing warrant exploration for stroke prevention [13]. This paper aims to provide an overview of the pathophysiology, classification, and management of stroke in patients with SCD, grounded in existing literature. Through this synthesized perspective, we seek to contribute to a holistic understanding of this topic and highlight opportunities for further research.

## Review

Methodology

Research Framework

This all-encompassing review is designed to shed light on the interconnections between SCD and stroke, explicitly emphasizing their pathophysiological links, classifications, and therapeutic interventions.

Database Exploration

A thorough examination of the scholarly databases PubMed, Scopus, and Cochrane was executed. Queries were made using key terms and synonyms such as "anemia," "stroke," "pathophysiology," "types," and "management," yielding a diverse array of pertinent studies.

Study Eligibility

For inclusion, research must focus on sickle cell anemia and stroke-like pathophysiology, types, or treatments to be published in English. Suitable study designs were randomized controlled trials (RCTs), observational studies, and various reviews. Exclusion criteria were non-scientific articles such as editorials and opinion pieces, as well as research with inadequate methodologies.

Analytical Overview and Data Integration

The studies that met the inclusion criteria were subjected to a comparative analytical review. This facilitated the identification of recurrent themes, variances, and unexplored areas within existing literature, offering a well-rounded view of sickle cell anemia's relationship to stroke.

**Figure 1:**
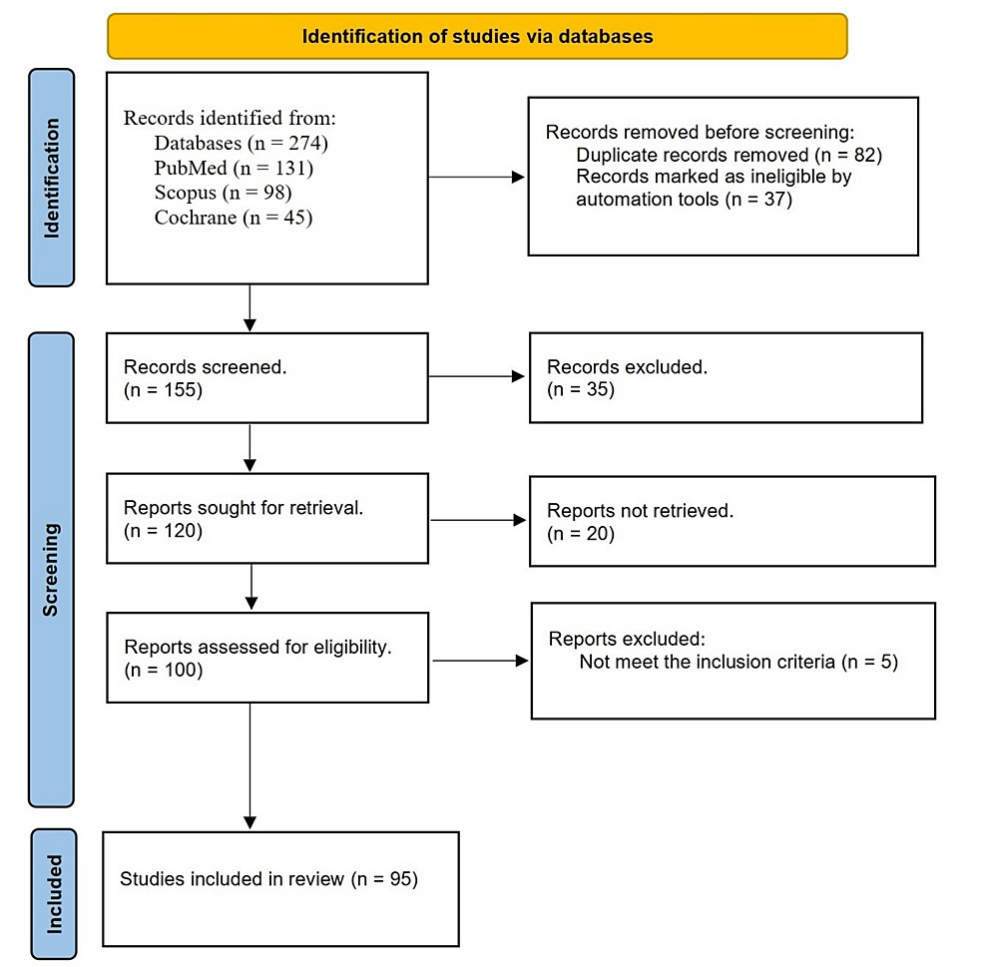
PRISMA flow diagram PRISMA: Preferred reporting items for systematic reviews and meta-analyses

*Study Selection Workflow* The study selection process is outlined in a flow diagram (Figure 1), following the preferred reporting items for systematic reviews and meta-analyses (PRISMA) guidelines. The initial search yielded 274 records. After duplicates were removed, 155 records remained. These records were screened based on their titles and abstracts, after which 35 records were excluded for not meeting the eligibility criteria. The remaining 120 full-text articles were evaluated to determine their suitability, and 25 articles were further excluded for various reasons, including not meeting the inclusion criteria, incorrect study design, and not reporting relevant outcomes. Finally, 95 studies were included for qualitative synthesis in the review.

Pathophysiology of SCD

Sickle cell disease originates from a single beta-globin gene mutation, producing abnormal hemoglobin S [14,15]. This protein prompts red blood cells to assume rigid, sickle shapes under low oxygen, causing vaso-occlusion by obstructing small blood vessels [16]. Vaso-occlusion results in ischemic injury and episodes of severe pain, known as sickle cell crises [17,18]. Sickled cells also cause chronic hemolytic anemia, resulting in oxidative stress and the impairment of endothelial function [15,19].

Recurrent sickling affects various organs, notably the spleen and brain [20,21]. In the spleen, it impairs immune function and raises infection risk [20]. In the brain, it can cause strokes, particularly in children [4,15]. Sickle cell disease pathobiology involves four core processes: (1) hemoglobin S polymerization; (2) vaso-occlusion; (3) hemolysis-induced endothelial dysfunction; and (4) sterile inflammation activation [18,22]. These mechanisms trigger erythrocyte injury, hypoxia-induced tissue damage, oxidant stress, and nitric oxide reduction [15,23].

Blockages in blood vessels (vaso-occlusive events) and the breakdown of red blood cells inside blood vessels (intravascular hemolysis) both fuel and drive inflammation in SCD [21,24]. Sterile inflammation is triggered by toll-like receptor 4 and inflammasomes, contributing to complications like chronic pain and end-organ failure [25,26]. Cardiovascular problems, including cardiomyopathy and pulmonary hypertension, result from chronic anemia, inflammation, and nitric oxide scavenging [27]. Neurological complications like cerebral ischemia and stroke also occur [12,21]. Overall, the disease involves complex pathophysiological processes, and while current treatments target key symptoms, more research is needed for targeted therapies [15,17].

Epidemiology, and morbidity of SCD

It is most prevalent in regions historically impacted by malaria, such as sub-Saharan Africa, Saudi Arabia, India, and the Mediterranean [28,29]. In Africa, where SCD manifests more severely, 240,000 children are born with the disease annually [29,30]. In the United States, SCD affects one in 365 African American births and one in 16,300 Hispanic American births [31,32]. The sickle cell gene, carried by up to 25% of the African population, offers some malaria protection, explaining its high frequency in malaria-endemic areas. While advances in care have increased life expectancy from 14 years in 1973 to over 40 years today, the disease remains a significant health challenge, especially in developing regions [28,32].

While mortality rates for SCD have declined dramatically over the past decades due to newborn screening and improved supportive care, they remain substantially higher than the general population [33,34]. The risk of death is highest in early childhood, with stroke and infections being common causes [34,35]. Morbidity rates are also very high, with most patients experiencing frequent pain crises, chronic anemia, recurrent infections, and organ damage early in life [36,37]. Over half of the patients have lung, liver, kidney, or heart impairment by age 18 [37,38]. Stroke is a significant complication, affecting 11% of patients by age 20 [4,39]. Acute chest syndrome (ACS) also frequently occurs, with a mortality rate of 2% to 4%. Other morbidities include retinopathy and priapism [40,41].

Pathophysiological association between SCD and stroke

Sickle cell disease causes red blood cells to be rigid and sickle-shaped [26,42]. These unusually stiff cells can become lodged in tiny blood vessels, thereby obstructing the circulation of blood [43]. When blood flow is blocked to any part of the brain, it causes a stroke. The lack of oxygen damages the brain tissue [11,26].

Red blood cells with a sickle shape have a higher propensity to adhere to the walls of blood vessels [43]. This makes the blood more viscous and slows blood flow, increasing the risk of clotting [44]. Clots can further block blood vessels and cause an IS. In addition, sickle cells have a shorter life span than normal red blood cells, leading to more cell debris and higher turnover [4,45]. This results in inflammation and the damage or weakening of blood vessel walls [46]. Weakened vessels are prone to rupture, which can block blood flow and cause stroke [45,47]. Chronic anemia leads to high cardiac output, increasing shear forces on blood vessel walls [48,49]. 

There is also increased activation of platelets in SCD, which promotes clot formation [24]. Chronic anemia leads to high cardiac output, increasing shear forces on blood vessel walls [48,49]. Children with SCD have a very high risk of stroke, estimated at 10% to 15% [4,50]. Without intervention, approximately 11% of patients suffer from clinical IS by age 20. Moreover, strokes tend to recur in these patients [4,50].

The IS is often due to stenosis or occlusion of major cerebral arteries like the internal carotid, middle cerebral, and anterior cerebral arteries [47]. Small vessel disease from sickling in capillaries and venules also increases the risk of IS as compared to the general population [12]. Areas of the brain with end arteries, like the subcortical white matter, deep gray matter, and watershed zones, are particularly vulnerable [51,52].

Sickle cell disease also causes intimal hyperplasia and the proliferation of smooth muscle cells in the arteries [8,53]. This thickens the vessel walls, narrowing their diameter and reducing blood flow [8,53]. Endothelial dysfunction is common too, which reduces nitric oxide and promotes vascular inflammation and thrombosis [53,54]. Abnormal interactions between sickle cells, endothelial cells, platelets, and coagulation factors further promote abnormal clotting [24,53].

The compensatory high-velocity blood flow in sickle cell anemia causes increased shear stress on vessels over time, damaging the endothelium [44,52]. Impaired vascular autoregulation makes it harder for the brain to adjust blood flow with pressure and oxygen changes [52,55]. Both significant artery stenosis and Moyamoya syndrome are observed; the latter causes fragile collateral vessels prone to rupture and hemorrhage [56]. Overall, both IS and HS are increased in SCD due to this multitude of vascular effects [4,50]. Blood transfusions can significantly reduce stroke risk by diluting sickle cell concentration [11,52].

Types of sickle cell hemoglobin associated with stroke

Sickle cell disease (SCD) encompasses various genotypes, with hemoglobin SS (homozygous for hemoglobin S), hemoglobin SC (compound heterozygous for hemoglobin S and hemoglobin C), and hemoglobin S beta-thalassemia being common examples. Hemoglobin SS is the most prevalent genotype associated with stroke in SCD (Table 1). Individuals with SS disease have the most significant risk of both IS and HS compared to other genotypes [57]. The SENET study found that the incidence rate of first stroke in children with SS disease was 0.61 events per 100 patient-years. In contrast, incidence was lower in SC disease (0.17/100 patient-years) and S-beta thalassemia (0.09/100 patient-years) [4].

**Table 1:** Summary of stroke risk in sickle cell disease by genotype HS: Hemorrhagic stroke; IS: Ischemic stroke

Genotype	Stroke risk comparison	Incidence rate (first stroke in children per 100 patient-years)	Notes	References
Hemoglobin SS	Highest risk of IS and HS	0.61 events	Most studied; considered a most significant risk	[4,57]
Hemoglobin SC	Lower risk than SS	0.17 events	Risk approaches that of SS when adjusted for confounders	[4,6]
S-beta thalassemia	Lower risk than SS	0.09 events	Risk lower than SS and SC, but still substantial compared to the general population	[4]
Other genotypes	Limited data	N/A	Studies are rare due to the rarity of these syndromes, often grouped with SS in studies	[58]

However, some studies suggest the relative risk of stroke in SC disease approaches that of SS disease when controlling for confounders like socioeconomic status and haplotype [6]. Data on stroke risk in other genotypes, like sickle-hemoglobin C disease, is more limited given the rarity of these syndromes. Most studies examining stroke pathogenesis and outcomes group all sickle cell genotypes together or focus only on SS disease. Few studies analyze differences between genotypes [58]. Small sample sizes make it difficult to draw definitive conclusions on relative stroke risk.

Overall, current evidence indicates SS disease carries the greatest stroke risk, while SC disease and S-beta thalassemia have a lower but still substantial risk compared to the general population. All patients with SCD should receive longitudinal screening and monitoring. More research is needed to determine if tailored stroke prevention strategies should differ based on specific genotypes. Standardization of care protocols will require collaborative studies across institutions.

Association between SCD and IS

Several studies have examined the epidemiology and risk factors for IS in SCD patients. Kawadler et al. found that the overall incidence of IS was 0.8 events per 100 patient years in a cohort of SCD patients from the United Kingdom [59]. The highest incidence occurred in early childhood, at five years of age [59]. Key risk factors for stroke identified across multiple studies include prior transient ischemic attack (TIA), low hemoglobin, high leukocyte count, and hypertension [60,61].

Neuroimaging techniques have been critical in understanding stroke mechanisms and outcomes in SCD. Magnetic resonance angiography (MRA) has shown that large-vessel intracranial stenosis is common but may or may not cause stroke [62,63]. Some propose a "two-hit" hypothesis where stenosis makes blood flow vulnerable to disruption and factors such as inflammation, sickling, or endothelial dysfunction trigger occlusion [62-64]. Silent cerebral infarcts detected on MRI (although many times they can also be detected by CT brain) are also associated with increased future stroke risk [6,64]. 

Numerous RCTs, including the stroke prevention trial in sickle cell anemia (STOP) and the transcranial Doppler (TCD) with transfusions changing to hydroxyurea (TWiTCH) trial, have assessed primary and secondary ischemic stroke prevention strategies [65,66]. Detailed discussions of these trials will be presented in the management and prevention sections.

While progress has been made, there are still knowledge gaps regarding exact stroke mechanisms and how to stratify stroke risk in SCD patients without neuroimaging. Overall, studies emphasize the importance of screening and continued research to improve preventative care and decrease stroke-related morbidity and mortality.

Association between SCD and HS

Hemorrhagic stroke is less common than IS in SCD but results in higher mortality rates. The exact prevalence of HS in SCD patients varies widely in studies, ranging from 1.3% to 11% [67,68]. This variation is due to differences in diagnostic methods, patient populations, and follow-up periods.

Intracerebral hemorrhage (ICH) appears to occur most frequently between the ages of 20 and 29 in SCD patients [69,70]. Hypertension is a significant risk factor, present in over half of SCD-ICH cases in some cohort studies [69-71]. Other identified risk factors include low hemoglobin, high reticulocyte count, prior ICH, and smoking [71,72].

The underlying pathophysiology of ICH in SCD is not entirely clear. Proposed mechanisms include vessel wall damage due to chronic anemia and sickling, as well as increased fragility of vessels from collagen depletion and smooth muscle hypertrophy [72,73]. Aneurysm formation related to vessel wall damage may also play a role. Neuroimaging studies have found that hemorrhages most often occur in the corticomedullary junction and lenticulostriate arteries, which are common locations of vascular collateralization and autoregulatory dysfunction [74,75]. This suggests that hemorrhages may originate from fragile collateral vessels.

There is no substantial evidence regarding acute ICH management specific to SCD patients. General guidelines include controlling blood pressure and considering surgery for extensive hemorrhages. The role of blood transfusions is debated due to concerns over increasing blood viscosity and exacerbating the hemorrhage [76,77].

Overall, many unknowns remain regarding risk factors and optimal treatment approaches for HS in SCD. Further research is needed through multicenter prospective studies to better characterize ICH incidence, establish screening protocols, and investigate novel therapies to improve prognosis. Standardized management protocols are also needed.

Prognosis of stroke in patients with SCD

Sickle cell disease patients confer substantially higher mortality and morbidity compared to the general pediatric stroke population [78,79]. Ischemic stroke, more common than HS in SCD, has relatively better short-term survival rates [80]. However, one major cohort study still found a 16% mortality rate after IS in SCD children [57]. Outcomes are worse with HS, with studies reporting mortality rates ranging from 40% up to a striking 62% [80]. For survivors, significant neurological deficits frequently persist. Motor impairments, such as hemiparesis, are particularly prevalent due to the extensive distribution of the motor cortex and subcortical pathways throughout the brain. The heightened risk of infarction in these areas can result in motor and coordination impairments, but language, cognitive, and behavioral deficits are also routinely seen [47,80]. One study found that 25% of children were left with severe residual disability after IS associated with SCD [57]. Without ongoing blood transfusions to reduce sickle hemoglobin, the risk of recurrent stroke is exceptionally high in SCD patients who have already suffered a stroke, with an approaching rate of 80% within just three years after the initial event [81,82]. Any cerebral injury, even radiographically silent infarcts detected on MRI, portends worse cognitive outcomes and elevated future stroke risk in SCD patients [6,82]. Furthermore, the prognosis is impacted by systemic barriers and socioeconomic disparities that reduce access to preventive care, acute treatment, and rehabilitation services in this historically marginalized population (Table 2) [78]. More research is critically needed to characterize long-term functional outcomes better, understand prognostic factors, and promote equity in care delivery to improve the poor prognosis of SCD patients after stroke.

**Table 2:** Summary of the prognosis of stroke in patients with SCD SCD: Sickle cell disease, HS: Hemorrhagic stroke

Outcome aspect	IS in SCD patients	HS in SCD patients	General notes and considerations	References
Mortality rate	16% in children	40-62%	High compared to the general pediatric stroke population	[57]
Recurrence rate	Up to 80% within three years	N/A	Requires ongoing interventions like blood transfusions to reduce risk	[81]
Neurological deficits	Frequent, including hemiparesis	Severe deficits frequent	Motor cortex and subcortical pathways are often affected; cognitive and behavioral issues are also common	[57]
Residual disability	25% of children were severely affected	N/A	Significant impact on long-term quality of life	[57]
Cognitive outcomes	Worse in the presence of silent infarcts	N/A	Even radiographically silent infarcts have negative cognitive implications	[6]
Long-term data availability	Limited	Especially limited for HS	Critical need for more research on long-term functional outcomes and prognostic factors	[78]
Impact of socioeconomic factors	Significant	Significant	Systemic barriers and disparities affect prognosis; access to care is crucial for improving outcomes	[78]

Clinical features of stroke in patients with SCD

Stroke represents one of the most severe complications associated with SCD, typically occurring in childhood. Ischemic stroke is more common than HS in SCD patients [4,83]. Major risk factors for IS include prior TIA, low hemoglobin, a high leukocyte count, and hypertension [59,84,85]. Hemorrhagic stroke has been associated with hypertension, low hemoglobin, a high reticulocyte count, and smoking [84,86]. Clinical features of IS in SCD patients are like those of non-SCD pediatric strokes. Common symptoms include hemiparesis, speech difficulties, and seizures [82,83]. However, some studies have found a higher frequency of bilateral motor deficits, presumably from multilobar infarction. Cranial nerve palsies and ataxia are also reported more often in SCD stroke patients [87].

Silent cerebral infarcts detected on MRI are common in SCD patients and are associated with cognitive deficits even without overt stroke symptoms [82,88]. Cognitive impairments after an overt stroke include issues with language, processing speed, memory, and executive functions [87,89]. However, long-term studies on cognitive outcomes are also lacking.

Clinical features of HS in SCD patients are typical of hemorrhages in other populations: acute severe headache, nausea, seizures, and focal neurological deficits based on location [86,90]. However, SCD patients tend to have smaller hematoma volumes than hypertensive ICH patients [85,90]. While many clinical features are like those of non-SCD strokes, some distinct patterns have been noted in SCD patients (Table 3).

**Table 3:** Summary of the clinical features of stroke in patients with SCD HS: Hemorrhagic stroke, SCD: Sickle cell disease, IS: Ischemic stroke, ICH: Intracerebral hemorrhage, TIA: Transient ischemic attack

Stroke type	Clinical features in SCD patients	Comparison with non-SCD strokes	Risk factors	Distinct SCD patterns	Additional notes	References
IS	Hemiparesis, speech difficulties, seizures	Similar to non-SCD pediatric strokes	Prior TIA, low hemoglobin, high leukocyte count, hypertension	Higher frequency of bilateral motor deficits, multilobar infarction	Silent cerebral infarcts associated with cognitive deficits	[85,90]
HS	Acute severe headache, nausea, seizures, focal neurological deficits	Typical of hemorrhages in the general population	Hypertension, low hemoglobin, high reticulocyte count, smoking	Smaller hematoma volumes than in hypertensive ICH	Cranial nerve palsies and ataxia are more common in SCD	[85,90]

Potential risk elements for stroke in patients with SCD

Multiple studies have identified key risk factors that amplify the likelihood of ischemic and HS in patients with SCD. For IS, the most commonly identified risk factors in various studies include prior TIA, with a specific emphasis on low hemoglobin levels and high leukocyte counts [57,85]. A cooperative study of SCD found that patients with a TIA had a 10-fold increased risk of subsequent IS compared to those without a TIA history [57,82]. Chronic transfusion programs after TIA have significantly reduced stroke risk, indicating TIA's importance as a warning sign [85,89,91].

Other potential risk factors include elevated TCD velocities, hypertension, ACS, and specific genetic modifiers, but study results are mixed on the strength of these associations [59,91]. Most studies examining genetics and stroke risk have been underpowered, given the complex multigenic nature of SCD [91]. For HS, the presence of hypertension appears to be the most significant risk factor, present in most patients [91,92]. Low hemoglobin, high reticulocyte counts, smoking, and excessive alcohol use have also been associated with increased risk [86,91].

Diagnosis of stroke in patients with SCD

Neuroimaging with CT or MRI is critical for determining stroke type and guiding management [90,93,94]. Several studies have evaluated imaging findings in SCD and other stroke patients. Both MRI and MRA are more sensitive than CT for detecting early cerebral infarcts and vascular abnormalities. Additionally, similar results from CT and MRI scans could be observed in comparison to other patients [90,94,95]. Silent cerebral infarcts detected by MRI in SCD patients are linked to a heightened risk of manifest stroke and neurocognitive impairment [6,90,95]. However, MRI availability is limited in many centers.

Transcranial Doppler ultrasound is not commonly used in clinical practice for screening and monitoring stroke risk. The American Society of Hematology (ASH) emphasizes the recommendation of annual TCD for children. Additionally, MRI and MRA are used in the screening of adult patients, particularly because clinically undetectable silent infarcts cannot be identified through standard clinical means [6,90].

Transcranial Doppler is a non-invasive and cost-effective alternative to MRI, yet it is operator-dependent and exhibits elevated false-positive rates [90,96]. Both methods share limitations, such as restricted accessibility in certain settings. Unfortunately, there is a scarcity of data supporting the use of TCD for assessing stroke risk in adults with SCD, and no standard recommendations currently exist [90,96]. In other stroke patients, TCD is electively used in non-acute settings for assessing individuals with TIA and IS to explore potential large-artery etiologies [97].

Advanced imaging techniques like perfusion-weighted MRI, positron emission tomography (PET), and functional MRI show promise for evaluating blood flow, metabolism, and functional reorganization in SCD stroke [51,90,98]. However, availability is limited, and definitive clinical roles are not established (Table 4).

**Table 4:** Summary of the diagnosis of stroke in patients with SCD SCD: Sickle cell disease, MRA: Magnetic resonance angiography, TCD: Transcranial Doppler, PET: Positron emission tomography, fMRI: Functional MRI

Diagnostic method	Key advantages	Key limitations	Notable findings and considerations	References
MRI and MRA	High sensitivity for cerebral infarcts and vascular abnormalities	Limited availability in some centers	Associated with increased risk of overt stroke and neurocognitive impairment	[6,90]
CT	Quick and widely available	Less sensitive than MRI/MRA, especially for early infarcts	CT use in older studies likely led to an underestimation of infarct prevalence	[90,94]
TCD ultrasound	Non-invasive, less expensive	Operator-dependent, high false-positive rates	Elevated blood flow velocities predict stroke risk; no consensus on optimal velocity cut-offs	[90,96]
Advanced imaging techniques (e.g., perfusion-weighted MRI, PET, fMRI)	Can evaluate blood flow, metabolism, and functional reorganization	Limited availability, no established definitive clinical roles	Show promise for future diagnostic and risk stratification; need further validation in collaborative studies	[51,90,98]

Stroke management in patients with SCD

The pathophysiological alterations that result in a stroke in a patient with SCD diverge from those in other stroke patients, necessitating distinct treatment approaches (particularly for IS). In SCD cases, the primary objective is to reduce hemoglobin S levels, a goal achievable through methods such as exchange transfusion or, albeit challenging to execute rapidly, simple blood transfusion [85,90,99,100].

For acute stroke management, the ASH guidelines recommend both hydroxyurea and transfusion treatment as primary strategies to prevent stroke in children diagnosed with SCD [47,90]. The choice between these two approaches depends on factors such as patient age, disease severity, availability of resources, patient preference, and potential risks and benefits. In conjunction with primary stroke prevention measures, management strategies for acute stroke in SCD include prompt recognition and treatment. Acute IS should be treated with intravenous thrombolysis using a tissue plasminogen activator (tPA) if eligible [90,101]. Mechanical thrombectomy may be considered in selected cases. Supportive care measures such as hydration, pain control, blood pressure management, and seizure prophylaxis are also necessary [102,103].

For HS, evidence on management in SCD is limited. Recommendations include controlling blood pressure and surgical evacuation for extensive hemorrhages [85,90]. However, the safety and efficacy of these interventions, specifically in SCD patients, need further study. A few studies have assessed hematopoietic stem cell transplants as a curative treatment for stroke. While feasible, it is associated with significant complications [104]. Transplants are likely best reserved for severe cases, given the risks [85,104]. Several knowledge gaps remain in optimal SCD stroke treatment. Small sample sizes and a lack of control groups have limited clinical trials. Many studies excluded patients with comorbid conditions, reducing generalizability. Standardized treatment protocols are needed to improve long-term outcomes. Overall, transfusions and hydroxyurea form the basis of current treatment. Clinical trials of novel therapies should be prioritized for all stroke patients, including those with SCD. Access to quality care remains a significant limitation and barrier to reducing stroke-related mortality.

Prevention of stroke in patients with SCD

Primary prevention of stroke in SCD centers around screening for risk and initiating transfusions in high-risk patients [66,105]. The STOP trial demonstrated a 90% reduction in stroke recurrence with regular transfusions compared to standard care. However, long-term transfusions carry a risk of iron overload [65]. For secondary prevention, the STOP II trial demonstrated that discontinuing transfusions led to a high rate of stroke recurrence or TIA. Continued indefinite transfusions are thus recommended after stroke but carry iron overload risks without chelation therapy [90,105].

The TWiTCH trial found hydroxyurea non-inferior to transfusion for secondary stroke prevention in pediatric SCD patients with abnormal TCD velocities [66,104]. Hydroxyurea may reduce stroke risk by decreasing sickle cell formation, but its role as primary prevention remains under study [66,105]. Chronic blood transfusions and hydroxyurea are primary prevention therapies, but the evidence is limited to the TWiTCH trial. The role of hydroxyurea in untreated SCD children requires further study.

Future research directions include refining stroke risk biomarkers, evaluating the effect of hydroxyurea starting in infancy, and determining the optimal duration of transfusions after stroke. Cost-effectiveness and implementation studies are also needed. Overall, while transfusions and hydroxyurea are beneficial, many questions remain about the optimization of stroke prevention strategies.

Rehabilitation of SCD patients after stroke

The management and rehabilitation of stroke in SCD patients are crucial for improving outcomes and reducing morbidity and mortality [85,106]. It is highly recommended to promptly begin opioid treatment for intense pain linked to vaso-occlusive crises as an acute complication management strategy [107,108]. Hydroxyurea is recommended for adults with recurrent vaso-occlusive crises or severe symptoms interfering with daily activities [85,90,107]. It is also suggested that hydroxyurea therapy be presented irrespective of symptoms to infants, children, and teenagers [85,107]. Long-term blood transfusion therapy is strongly recommended for preventing strokes in children with abnormal TCD velocity [108].

Cognitive rehabilitation programs have been piloted to improve memory and educational achievement in children with SCD who have had strokes [85,108,109]. Although these interventions are feasible, additional research is needed to establish their efficacy. Ongoing studies are designed to improve cognitive dysfunction in children diagnosed with SCD and stroke [107,109]. Exercise has been shown to favor cardiovascular remodeling in hypertrophic cardiomyopathy (HCM) patients [106,110]. Moderate exercise programs do not present any safety issues for patients with HCM. Additionally, people with HCM face similar risks from conditions like atherosclerosis as the general public [108]. Studies have linked exercise to a lower risk of heart attack, stroke, and heart failure in this population [106,108]. Regular physical activity may help reduce the threats posed by these atherosclerotic risk factors for individuals with HCM [106,110].

There is a concerning lack of research focused explicitly on rehabilitation strategies and outcomes for SCD patients who have suffered a stroke [108]. Most published literature consists only of small case reports or retrospective series with no control groups, limiting the ability to conclude. No RCTs have been conducted to date evaluating different rehab approaches for this population. Initial studies suggest SCD patients may achieve functional gains with standard inpatient rehabilitation programs but likely have worse outcomes compared to pediatric stroke patients without SCD [106,108]. Isolated cases have trialed interventions like constraint-induced movement therapy with success, but no robust evidence exists for any specific protocols. Furthermore, research on cognitive rehabilitation is completely lacking despite frequent impairments after SCD stroke [89,106,108]. Significant access barriers to obtaining intensive services also persist for these patients due to socioeconomic factors.

## Conclusions

Sickle cell disease poses a major risk for debilitating IS and HS, especially in childhood. The pathophysiology involves sickling-induced vascular dysfunction, damage, and occlusion. While transfusions and hydroxyurea reduce stroke recurrence, primary prevention and acute treatment remain suboptimal. Neuroimaging helps distinguish stroke types, but protocols need standardization. Though IS is more common, HS has higher mortality in this population. Significant knowledge gaps persist regarding exact stroke mechanisms, stratifying risk, optimizing screening, improving access to care, and managing long-term outcomes. A high index of suspicion for stroke should be maintained when new neurological symptoms emerge in SCD patients, given the high risk. Also, high-quality research and an emphasis on equitable access are critically needed to optimize care and improve outcomes for SCD patients after stroke. Rehabilitation studies specific to this population should be prioritized.

Overall, this review highlights opportunities for further research and establishing evidence-based protocols across institutions to mitigate the alarmingly high risk of stroke in patients with this chronic hematologic disorder. A holistic perspective encompassing pathophysiology, screening (with a specific focus on patients with prior TIA, low hemoglobin, elevated leucocytes, and high blood pressure), prevention, acute treatment, rehabilitation, and socioeconomic factors is critical for improving the traditionally poor prognosis of strokes in children and adults with SCD.
